# Supplementary material for: Pipelined and conflict-free number theoretic transform accelerator for CRYSTALS-Kyber on FPGA
Source: PLoS One. 2025 Nov 11;20(11):e0333301. doi: 10.1371/journal.pone.0333301 (PMC12604810; doi:10.1371/journal.pone.0333301)
Supplement: Attachment — Submitted filename: Response to Reviewers.docx [file pone.0333301.s002.docx]

**Editor’s and Reviewer’s Comments**

The Authors would like to take this opportunity to appreciate Editor and Reviewers Constructive feedback and made sure that all the comments made by the worthy persons are addressed to the best of Author’s knowledge. Please find below the Authors response to the Editor/Reviewer comments. These are also being incorporated in the revised manuscript uploaded by the Authors.

| **Editor/Reviewers** | **Comments** | **Response** |
| --- | --- | --- |
| **Editor Comments** | 1. Please ensure that your manuscript meets PLOS ONE's style requirements, including those for file naming. | The Authors have updated the manuscript to meet PLOS ONE’s style requirements. |
|  | 2. Please note that PLOS ONE has specific guidelines on code sharing for submissions in which author-generated code underpins the findings in the manuscript. In these cases, we expect all author-generated code to be made available without restrictions upon publication of the work. | The Authors acknowledge PLOS ONE's code-sharing guidelines and confirm that the author-generated code will be made available without restrictions upon acceptance |
|  | 3. When completing the data availability statement of the submission form, you indicated that you will make your data available on acceptance. We strongly recommend all authors decide on a data sharing plan before acceptance, as the process can be lengthy and hold up publication timelines. Please note that, though access restrictions are acceptable now, your entire data will need to be made freely accessible if your manuscript is accepted for publication. This policy applies to all data except where public deposition would breach compliance with the protocol approved by your research ethics board. If you are unable to adhere to our open data policy, please kindly revise your statement to explain your reasoning and we will seek the editor's input on an exemption. Please be assured that, once you have provided your new statement, the assessment of your exemption will not hold up the peer review process. | The Authors comply with PLOS ONE’s data-sharing requirements and confirm that we are aware that code sharing is mandatory. Upon acceptance, we will make the code freely accessible in a public repository, in full compliance with PLOS ONE’s conditions. |
|  | 4. If the reviewer comments include a recommendation to cite specific previously published works, please review and evaluate these publications to determine whether they are relevant and should be cited. There is no requirement to cite these works unless the editor has indicated otherwise. | After reviewing the recommended works, the Authors found them to be relevant to our study. Accordingly, the suggested previously published work has been incorporated into the revised manuscript. |
| **Reviewer # 1 Comments** | 1. Table II is not complete; add results for throughput per unit area, like throughput per slice. Also comment on the power consumption of the design. | As per the Reviewer suggestion, results of throughput per slice have been added in **Table 2,** **section** **“Result analysis”, subsection “NTT comparison”, Page #20.**  Additionally, the power figures have been added in **section** **“Result analysis”, on Page #24, Line #761-762.** |
|  | 2. References are used in a very strange manner, like (1) and (2). This representation is used for equations, not for references. | The Authors appreciate the Reviewer’s insights and have revised the manuscript to ensure that all references are now correctly formatted according to the required citation style. |
|  | 3. A lot of typos and grammatical mistakes; it is advised to proofread the manuscript very carefully. In addition, a few lines of paragraphs are not recommended, so reduce the number of paragraphs by increasing their sizes. | The Authors are thankful to the Reviewer for their valuable feedback. The Authors have thoroughly proofread the manuscript and corrected all typos and grammatical errors. Additionally, shorter paragraphs have been reviewed and where appropriate, merged to form more cohesive and comprehensive paragraphs for improved readability and flow. |
|  | 4. The authors must explain whether it is in-place NTT or any other form. How many BRAMs are used to store the polynomial coefficients, and is it for a single polynomial? | An in-place NTT implementation stores the coefficients in BRAMs or registers and updates them directly at each stage of the butterfly computation. It implies, instead of reading from one memory array and writing results to another, the same memory location is reused.  The proposed NTT design is based on a **Single-Path Delay Feedback (SDF)** architecture, which differs from a classical in-place implementation. In our design, the polynomial coefficients are streamed through seven butterfly stages. Each stage uses **dedicated FIFOs** for memory handling and data alignment, and intermediate values are not stored in the same memory locations. Hence, this architecture does **not follow the traditional in-place pattern** but rather adopts an out-of place NTT approach **with stage-wise dataflow** for efficient pipelining and resource utilization.  Moreover, our architecture is designed to be BRAM-free and instead uses FIFO buffers to stream polynomial coefficients. The design handles one polynomial at a time, and intermediate values are passed between the stages using dedicated FIFOs associated with each butterfly unit. This approach allows for a compact and efficient dataflow without the need for block RAM storage.  The discussion is added in revised Manuscript **section** “**Proposed architecture” subsection “Twiddle factors storage and management”, on Page #15, Line #453-465.** |
|  | 5. The authors didn’t mention their previous work, ‘Area-time efficient pipelined number theoretic transform for CRYSTALS-Kyber’, is there any good reason? | The Authors appreciate the Reviewer for highlighting this point. We have now included our previous work, “Area-time efficient pipelined number theoretic transform for CRYSTALS-Kyber,” in the revised manuscript **section** **Literature Review”, on Page #5, Line #151-153.** |
|  | 6. Provide your modular multiplier, butterfly unit results, and compare them with: (1) ‘efficient reconfigurable modular multipliers for post-quantum digital signatures’, and (2) ‘efficient soft-core multipliers for digital signatures’. Add this comparison to the manuscript. | The Authors appreciate the valuable suggestions of the Reviewer, and new subsections **“Butterfly unit comparison”** and **“Modular multiplier comparison”** are added in revised manuscript, **section “Result Analysis**”, for comparison of our butterfly unit and modular multiplier unit with state of the art architectures. These subsections are added for clear readability and understanding.  Paper (1) ‘efficient reconfigurable modular multipliers for post-quantum digital signatures’ adopts an interleaved multiplication approach for modular multiplication. The complete ATP and TP/A comparison of our proposed modular multiplier with paper (1) and other prior works is given in Table 4, sub section **“Modular multiplier comparison”, on page #23-24.** The comparison analysis of paper (1) with our proposed modular multiplier is given in sub section **“Modular multiplier comparison”, on page #23, Line #730-733**.  Paper (2) ‘efficient soft-core multipliers for digital signatures’ presents PM (5x5 multiplier without reduction) and PFFM (5x5 multiplier with reduction) for post quantum digital signature scheme MAYO with q=31. Our proposed multiplier is a **12×12 modular multiplier for CRYSTALS-Kyber** (q = 3329). To facilitate a fair comparison of our core with paper (2) we instantiated our modular multiplier with a 5-bit operand. The comparison is given in **sub section “Modular Multiplier comparison”, on page #24, Line #747-757.**  Paper (1) and paper (2) only propose modular multipliers hence the authors didn’t include them in butterfly unit comparison. However, the complete result analysis of our proposed butterfly unit with state-of-the-art architectures is given in **sub-section “Butterfly unit comparison”, on Page #22-23, Line #702-720.** |
|  | 7. Some recent work must be added and compared in Table 2, such as (1) Efficient Number Theoretic Transform Architecture for CRYSTALS-Kyber, etc. | As per the Reviewer’s comments, the Authors have added the suggested paper in Table 2 for comparison with the proposed work. The analysis of ATP comparison of suggested paper with our work is given in section **“Result Analysis”,** **sub-section “NTT comparison”, on Page #21, Line #678-682.** |
|  | 8. Most of the schemes are now hybrid, so how will your design scale in a multi-scheme environment where lattice and hash-based schemes are integrated? | The authors appreciate the Reviewer’s observations on the scalability of our design within a multi-scheme environment. Notably, CRYSTALS-Kyber and CRYSTALS-Dilithium employ distinct prime modulus parameters, with q values of 3329 and 8,380,417, respectively. Scalability can be achieved by developing a unified butterfly architecture capable of handling both q=3329 and q=8,380,417. Through such unified butterfly units, the proposed SDFNTT architectures can efficiently perform polynomial multiplications for both Kyber and Dilithium. The above discussion is added to a new **sub-section “SDFNTT for Dilithium”** in the revised manuscript under **section “Proposed Architecture” page # 18 line # 584-588**  Although this paper primarily presents a Kyber-specific implementation, hybrid designs have been identified as future research directions and are outlined in the section **“Future Work”, on Page #25, Line #794-796.** |
|  | 9. Power consumption is very important; add power consumption stats and compare them with the listed designs. | The Authors appreciate the suggestion of the Reviewer and have added the power figures in the revised manuscript **section “Result Analysis”, sub-section “Resource utilization analysis” on Page #24, Line #763-764.**  The Authors would like to clarify that the designs listed in Table 2 of the manuscript do not provide power consumption figures in their publications. Therefore, a direct power comparison with those works was not possible. |
|  | 10. Side-channel attacks are very important nowadays. How robust is your design against these attacks (power and timing)? | The authors acknowledge the importance of side-channel analysis in cryptographic systems. Our architecture executes operations in constant time and therefore is inherently resistant to timing-based side-channel attacks (SCAs). The discussion is added in **section “Conclusion”, on Page #25, Line #781-786.**  While the current work only focuses on area-time efficiency, the future work will investigate countermeasures against other forms of SCAs, such as power analysis attacks and fault-injection-based attacks. This is mentioned in Revised Manuscript, **section “Future Work”, on Page #25, Line #798-800.** |
| **Reviewer # 2 Comments** | 1. When a new implementation is proposed it must be analyzed from side-channel aspects. It is suggested that the authors explain the potential side-channel leakages of their architecture (e.g., timing, power)? If there are any, do they have considered any potential countermeasures (e.g., constant-time logic, masking, etc.)? | The Authors thank the reviewer for this important comment. The Authors acknowledge that side-channel analysis (SCA) is highly relevant in evaluating cryptographic hardware. Our proposed architecture executes in constant time, which inherently provides resistance against timing-based SCAs. This is mentioned in revised manuscript, **section “Conclusion”, on Page #25, Line #781-786.**  However, we recognize that other types of side-channel leakages, such as power analysis or fault-injection-based attacks are possible in hardware designs. The current work did not incorporate dedicated countermeasures such as masking, hiding or noise injection, as our primary focus was on achieving area–time efficient implementations.  We agree with the reviewer that developing countermeasures against these attacks is necessary. Therefore, our future work will focus on integrating countermeasures against power-based and fault-based SCAs to strengthen robustness while maintaining efficiency for lightweight PQC implementations. This is mentioned in Revised Manuscript, **section “Future Work”, on Page #25, Line #796-798.** |
|  | 2. It is beneficial to add a discussion and explain the challenges of adopting the proposed approach to other LWE schemes that use NTT such as Dilithium. | The authors appreciate the reviewer’s valuable insight. Kyber and Dilithium are both lattice-based schemes, where Kyber is a key encapsulation mechanism (KEM) and Dilithium is a digital signature scheme. Both rely on the NTT for polynomial multiplication.  The key difference lies in their modulus sizes: Kyber uses a 12-bit modulus (q = 3329), while Dilithium employs a 23-bit modulus (q = 8380417). This implies that Kyber requires narrower datapaths, whereas Dilithium demands significantly larger bit-widths when implemented in hardware.  The proposed SDF-based NTT architecture for Kyber can be adapted to support the Dilithium scheme by designing a butterfly architecture with wider bit-widths and integrating a modular multiplier for the larger modulus q=8380417. This demonstrates the scalability of our architecture across different lattice-based cryptographic schemes.  Kyber is not NTT-friendly for Negative wrapped convolution (negacyclic), as its modulus q=3329 does not support a primitive 2n-th root of unity. Therefore, Kyber adopts a truncated NTT approach, which requires only 7 stages of butterfly units for n=256. In contrast, Dilithium’s modulus q=8380417 is NTT-friendly, enabling a full negacyclic NTT. Consequently, a Dilithium SDFNTT implementation will require the complete 8 stages of butterfly units.  A new **sub-section “SDFNTT for Dilithium”** is added in the Revised Manuscript for the above discussion, in section **“Proposed Architecture” on page#18-19, line #575-584, #589-593** |
|  | 3. Please also add power and energy metrics of your design. | The power and energy metrics are added in revised Manuscript, **section “Result Analysis”, sub-section “Resource utilization analysis” on Page #24, Line #763-766.** |
|  | 4. Please mention the synthesis and implementation settings that you used on Vivado. Also, there are several typos and errors throughout the manuscript. Please proofread the whole manuscript several times | The Authors appreciate the valuable suggestion of the Reviewer and would like to mention that both synthesis and implementation were performed under the default Vivado settings. The same is also added in Revised Manuscript **section “Result Analysis”, on page #19, line #599-600.**  Additionally, the Authors have carefully proofread the entire manuscript multiple times and corrected typographical and grammatical errors to ensure clarity and readability. |
|  | 5. Picture qualities are bad especially when zoomed. It is suggested that the authors import the pdf file of their figures instead of jpeg, png, etc. | We thank the reviewer for this helpful suggestion. In the revised manuscript, all figures have been updated to higher quality. Specifically, we have used the **PLOS online image preparation tool** PACE to convert the figures into **TIFF format,** ensuring that they maintain clarity and resolution when zoomed. This improves readability and ensures the figures meet publication standards. |
|  | 6. Eq 1?? | The Authors have corrected the format of numbering of equation 1 in the revised manuscript, **section “Proposed architecture” sub-section “Modular reduction unit”, on page #11, line #337** |
|  | 7. CRYSTALs must be CRYSTALS | The Authors have changed CRYSTALs to CRYSTALS in the entire document. |
|  | 8. (8),when (no space) | The Authors have added space between (8), and when, **section “Preliminaries” sub-section “Classical NTT vs NTT in Kyber”, on page #5, line #157.** |
|  | 9. [52] | Reference [52] was previously cited twice: once in the **subsection “Integer Multiplier”** and once in the **subsection “Modular Reduction Unit”**. Upon careful review, the authors identified that the citation in the **“Integer multiplier” subsection** was not relevant and have therefore removed it. The remaining citation in the “**Modular reduction unit” subsection, on page#9, line #287**, has been retained and is, to the best of the authors’ knowledge, correctly formatted. |
|  | 10. Analysis show(s) | The Authors have corrected the grammatical error in the revised manuscript, **section “Proposed architecture”, sub-section “NTT comparison”, page #21, line #669.** |
|  | 11. An acronym must be defined only once when it is used for the first time. From there it must be redefined again. Do this to all the acronyms you use, including ENS. | The Authors have carefully revised the manuscript to ensure that each acronym is defined only once at its first occurrence (including ENS and all others). |
